# Supplementary material for: Bio-Based Polymer Developments from Tall Oil Fatty Acids by Exploiting Michael Addition
Source: Polymers (Basel). 2022 Sep 28;14(19):4068. doi: 10.3390/polym14194068 (PMC9571392; doi:10.3390/polym14194068)
Supplement: Supplementary file 1 [file polymers-14-04068-s001.zip › polymers-1919468-supplementary.pdf]

polymers MDPI

Table S1. Characteristics (glass transition temperatures (T_g) by DMA, tensile (Young's) modulus (E^1), tensile strength and cross-link density (ν_e)) of selected polymers for comparison.

Materials(raw materials, short preparation method)	Samples	Characteristics				Ref.
		T_g (DMA), °C	E', (MPa)	Tensile strength, MPa	ν_e, mol·cm^{-3}	
On the basis of acetoacetylated soybean oil (AASBO), several bio-based coating materials were prepared using different aromatic dicarboxaldehydes (1,2-benzenedialdehyde (1,2-BDA), 1,3-benzenedialdehyde (1,3-BDA), 1,4-phthalaldehyde (1,4-BDA), 4,4′-biphenyldicarboxaldehyde (4,4′-BPDCA))	AASBO with 1,2-BDA	38	2.76	1.44	$1.4\cdot10^{-5}$	[39]
	AASBO with 1,3-BDA	39	6.27	3.54	$3.5\cdot10^{-5}$	
	AASBO with 1,4-BDA	42	15.07	3.85	$8.9\cdot10^{-5}$	
	AASBO with 4,4′-BPDCA	54	24.91	5.65	$9.0\cdot10^{-5}$	
Film materials prepared from acetoacetylated castor oil or modified castor oil (modified by different amount of 2-mercaptoethanol (Castrol oil:2-mercaptoethanol: 0.01:0.015 and 0.01:03)) and 4,4-diaminocyclohexylmethane (PACM) by Michael addition reaction	Film from acetoacetylated castor oil and PACM	2	0.028	0.68	$0.097\cdot10^{-3}$	[43]
	Film from modified castor oil (0.01:0.015) and PACM	27.6	0.041	1.06	$0.17\cdot10^{-3}$	
	Film from modified castor oil (0.01:0.015) and PACM	33.1	0.18	1.75	$0.27\cdot10^{-3}$	
Polyurethane (PU) films prepared from epoxidized soybean oil (ESBO) and epoxidized linseed oil (ELO) ring-opened by polyhydroxy fatty acids. Polyols were identified as SMS, SGS, LMS, and LGS, where the first letter "S" – ESBO, "L" – ELO; the second letter "M" – methanol , "G" – glycol; "S" refers to ESBO ring-opened by fatty acids.	SMS-PU	39.3	67.2	8.6	$0.0374\cdot10^{-3}$	[5]
	SGS-PU	49.9	123.4	11.5	$0.103\cdot10^{-3}$	
	LMS-PU	63.4	166.6	13.5	$0.31\cdot10^{-3}$	
	LGS-PU	80.5	315	17.2	$0.91\cdot10^{-3}$	
Bisphenol A epoxy resin was modified with a dimeric fatty acid and diglycidyl groups. The prepared polyol was mixed with polypropylene glycol and reacted with isophorone diisocyanate	PU	108	2035	~68	$1.03\cdot10^{-3}$	[52]
Hydroxyl-terminated fourth-generation hyperbranched polyester was synthesized from glycerol, 2,2-bis(Hydroxymethyl) propionic acid, 3-isocyanatopropyl triethoxysilane	PU	116.6	293		$2.97\cdot10^{-3}$	[53]
Vinylogous urethane vitrimer derived from renewable castor oil and DL-limonene	PU vitrimers	48	27.2	5.5	$0.99\cdot10^{-3}$	[74]
Non-isocyanate polyurethane (NIPU) prepared from hexamethylene diamine and glycerol cyclic carbonates, trimethylolpropane	NIPU		2100	68		[15]

**Materials(raw materials, short preparation method)**	**Samples**	**Characteristics**				**Ref.**
		**T_g (DMA), °C**	**E', (MPa)**	**Tensile strength, MPa**	**ν_e, mol·cm^{-3}**	
NIPU prepared by curing carbonated soybean (CSBO) and linseed (CLSO) oils with different diamines (1,2-ethane diamine (EDA), 1,4-butane diamine (BDA), isophorone diamine (IPDA))	NIPU (CSBO-EDA)	20	6	4		[75]
	NIPU (CSBO-BDA)	17	2	2		
	NIPU (CSBO-IPDA)	40	50	5		
	NIPU (CLSO-EDA)	55	180	18		
	NIPU (CLSO-BDA)	45	300	17		
	NIPU (CLSO-IPDA)	60	1460	10		
Methyl methacrylate (MMA)	Poly(methyl methacrylate) (PMMA)		2700	55		[68]
MMA	PMMA	126.5	1514		0.081·10^{-3}	[56]
